# ASO Author Reflections: The Association Between Sentinel Lymph Node Biopsy and Melanoma-Specific Survival in the Elderly

**DOI:** 10.1245/s10434-024-15785-w

**Published:** 2024-07-26

**Authors:** Hanna Kakish, Luke D. Rothermel

**Affiliations:** University Hospitals Cleveland Medical Center, Cleveland, OH USA

## PAST

Sentinel lymph node biopsy (SLNB) for melanoma is used less frequently in elderly patients.^1^ SLNB provides crucial prognostic information for adult patients with clinically localized melanoma based on the Multicenter Selective Lymphadenectomy Trial-I (MSLT-I); however, patients older than 75 years of age were excluded from this trial.^2^ Improved melanoma-specific survival (MSS) was seen in a latent subgroup analysis of the MSLT-I for patients with intermediate-thickness tumors whose nodal disease was discovered through SLNB rather than after a clinical recurrence.^2^ In melanoma patients older than 75 years of age, institutional data from Sabel et al. (1996–2011) reported a trend toward improved MSS in patients undergoing SLNB versus those not undergoing SLNB.^3^ To date, the prognostic and therapeutic benefits of SLNB in elderly patients with melanoma remains debated.

## PRESENT

In the current analysis of the Surveillance, Epidemiology, and End Results (SEER) dataset,^4^ we describe a persistently high rate of nodal positivity across elderly age groups (≥70 years of age) with T2-4 melanomas. Even so, increasing age was associated with decreased SLNB performance. A negative SLNB was independently associated with improved MSS compared with those who did not undergo SLNB, for patients up to 87 years of age. A positive SLNB was independently associated with inferior MSS compared with those who did not undergo SLNB, across all age groups. These results affirm the prognostic value of SLNB in the elderly and show a persistent association between SLNB performance and MSS across all age groups. The survival benefit was neither diminished nor eliminated in the elderly population. Therefore, SLNB should not be omitted based on age alone if patients can otherwise tolerate surgery.

## FUTURE

Questions remain about whether SLNB informs treatment decisions and improves oncologic outcomes, or whether SLNB use should be de-escalated in elderly populations. Future clinical trials should include elderly patients with melanoma to assess the association of SLNB with the utilization of adjuvant therapies, recurrence-free survival, and MSS.
